# E7 proteins from oncogenic human papillomavirus types transactivate p73: role in cervical intraepithelial neoplasia

**DOI:** 10.1038/sj.bjc.6600033

**Published:** 2002-01-21

**Authors:** L A Brooks, A Sullivan, J O'Nions, A Bell, B Dunne, J A Tidy, D J Evans, P Osin, K H Vousden, B Gusterson, P J Farrell, A Storey, M Gasco, T Sakai, T Crook

**Affiliations:** Ludwig Institute for Cancer Research, St Mary's Hospital Medical School, Norfolk Place, London W2 1PG, UK; University Department of Pathology, Glasgow University, Western Infirmary, Glasgow, UK; Department of Gynaecological Oncology, University of Sheffield, Northern General Hospital, Sheffield, S5 7AU, UK; Department of Histopathology, St Mary's Hospital Medical School, Norfolk Place, London W2, UK; Department of Pathology, University College Hospital, London, WC1, UK; ABL Basic Research Program, NCI-Fredrick Cancer Research and Development Center, Fredrick, Maryland, MD 21702-1201, USA; Centre for Cutaneous Research, St Bartholomew's and the Royal London School of Medicine and Dentistry, London E1 2AT, UK; UO Oncologia Medica, Azienda Ospedaliera S. Croce e Carle, Via Coppino 26, 12100, Cuneo, Italy; Department of Preventitive Medicine, Kyoto Prefectural University of Medicine, Kawaramachi-Hirokoji, Kamigyo-ku, Kyoto 602-8566, Japan

**Keywords:** HPV, p73, cervical cancer

## Abstract

In common with other E2F1 responsive genes such as p14^ARF^ and B-*myb*, the promoter of p73 is shown to be positively regulated in cell lines and primary human keratinocytes by E7 proteins from oncogenic human papillomavirus (HPV) types 16, 18, 31 and 33, but not HPV 6. Mutational analysis revealed that transactivation of the p73 promoter by HPV 16E7 requires association with pRb. Expression of p73 in normal cervical epithelium is confined to the basal and supra-basal layers. In contrast, expression in neoplastic lesions is detected throughout the epithelium and increases with grade of neoplasia, being maximal in squamous cell cancers (SCC). Deregulation of expression of the N-terminal splice variant p73Δ2 was observed in a significant proportion of cancers, but not in normal epithelium. The frequent over-expression of p73Δ2, which has recognized transdominant properties, in malignant and pre-malignant lesions suggests a role in the oncogenic process in cervical epithelium.

*British Journal of Cancer* (2002) **86**, 263–268. DOI: 10.1038/sj/bjc/6600033 www.bjcancer.com

© 2002 The Cancer Research Campaign

## 

p73 has structural and functional homologies to p53, including sequence-specific DNA binding and transactivation (Kaghad *et al*, 1997). Over-expression of p73 is able to induce apoptosis in some human cancer lines (Jost *et al*, 1997) and expression of p73 has been shown to have a role in the differentiation of keratinocytes (De Laurenzi *et al*, 2000). p73 exists in a number of isoforms which arise by alternative splicing of exons encoding the −COOH terminus of the protein and which exhibit differences in trans-activating and growth suppressor functions (De Laurenzi *et al*, 1998). Expression of a transdominant variant lacking exon 2 (p73 Δ2) has been described in ovarian cancer (Ng *et al*, 2000) and in breast cancer cell lines (Fillippovich *et al*, 2001). Recently, p73 has been shown to be directly induced by E2F1 and thereby to contribute to E2F1-mediated apoptosis (Irwin *et al*, 2000; Lissy *et al*, 2000). Furthermore, both myc and adenovirus E1A can activate expression of p73 (Zaika *et al*, 2001).

Although p73 is subject to methylation-dependent transcriptional silencing in some B-cell malignancies, consistent with a role as a putative tumour suppressor protein (Corn *et al*, 1999), mutational analyses have suggested that it is rarely mutated in human cancers. Furthermore, p73 is over-expressed in some cancers, although the mechanism of this is unknown (Chi *et al*, 1999; Zaika *et al*, 1999).

Squamous cell carcinoma of the cervix is the second most common female cancer. The majority of cervical SCC contain and express sequences from oncogenic HPV types, principally HPV 16, 18 and 31. Two proteins, E6 and E7, encoded by oncogenic HPV types have critical roles in carcinogenesis. E7 is able to associate with and functionally inactivate the protein (pRb) encoded by the *RB* gene. Expression of both E6 and E7 is necessary for immortalization of primary genital keratinocytes (Hawley-Nelson *et al*, 1989), implying that continued expression of HPV-encoded oncoproteins will be fundamental to growth of HPV positive cancer cells.

The association of E7 with pRb causes release of E2F1 from complexes with pRb resulting in expression of a number of genes with critical functions in the control of cellular proliferation including p14^ARF^, the alternative product of the INK4 locus (Bates *et al*, 1998) and B-*myb* (Lam *et al*, 1994). Expression of p14^ARF^ is hypothesized to protect cells from oncogenic events by inhibiting MDM2-dependent degradation of p53, facilitating p53-induced apoptosis and cell cycle arrest (Bates *et al*, 1998). Induction of p14^ARF^ expression by E2F1 is consistent with such a function, since expression of other oncoproteins, such as adenovirus E1A and SV40 T, also results in deregulation of E2F1 (Zaika *et al*, 2001). In this study, we have investigated the deregulation of p73 by HPV E7, both *in vitro* and in clinical biopsies positive for HPV 16.

## MATERIALS AND METHODS

### Transient assays

H1299 cells were maintained in DMEM with 10% foetal bovine serum. Primary human keratinocytes were purchased from Clonetics and grown according to the manufacturer's instructions. H1299 cells were transfected by the calcium phosphate method and primary keratinocytes using Effectene (Qiagen) following the manufacturer's instructions. Transfection efficiency was controlled by inclusion of pSV β-gal in each assay. Luciferase activity was determined using the Promega assay system and normalized to β-galactosidase activity. The reporter plasmids were p73 luciferase (Ding *et al*, 1999), p14^ARF^ luciferase (Bates *et al*, 1998) and B-*myb* luciferase (Lam *et al*, 1994). Each transfection included 1 μg of reporter. HPV 16E7 expression plasmids were constructed by sub-cloning into pcDNA3 inserts from pMo16E7, pMo24Gly, pMo26Gly and pMoΔ21-35 (Edmonds and Vousden, 1989). Plasmids for expression of E7 from other HPV types were constructed by sub-cloning the inserts from pJ4Δ E7 plasmids previously described into pcDNA3 (Storey *et al*, 1989). The adenovirus expression plasmid was pE1A (Zaika *et al*, 2001).

### Cervical tissue specimens

Cervical carcinomas, cervical intraepithelial neoplasia (CIN) tissues and matched, normal cervical epithelium were obtained at hysterectomy or cone biopsy. In each case the diagnosis was checked by histopathological analysis, and a majority of neoplastic cells in the tissue for analysis was confirmed. Tissues were collected immediately into liquid nitrogen and stored until analysis. Genomic DNA was isolated by proteinase-K digestion and total RNA by RNAzol B. HPV DNA sequences were sought by in all specimens using type specific PCR for HPV types 16, 18, 31, 33 and 39 as described by Walboomers *et al*. (1999). Paraffin sections of cervical neoplasia for immunocytochemistry were retrieved from the Department of Histopathology at St Mary's Hospital, London.

### Analysis of gene expression of p14^ARF^ and p73

cDNA was synthesised with the ProStar system (Stratagene) from 3 μg of total RNA. Analysis of expression was performed by RT–PCR as described previously for p14^ARF^ (Gazzeri *et al*, 1998), and p73 (De Laurenzi *et al*, 1998). For semi-quantitative analysis of gene expression, PCR was performed using the primers and thermal cycling conditions described and was for 22 cycles for p14^ARF^, and 28 cycles for p73. In some experiments, amplification was extended to 40 cycles to analyze expression in tissues expressing a lower level of p73. Following PCR, reactions were resolved on agarose gels, transferred to Hybond-N^+^ nylon and hybridized with ^32^P γ-ATP-labelled oligonucleotide probes specific for the amplified fragments. Analysis of N-terminal splice variants of p73 was performed using the primers described (Ng *et al*, 2000). Identity of these was confirmed by cloning and sequencing and by hybridization analysis of amplified products with oligonucleotide probes specific for exon 2 and exon 3 of p73. The presence of equivalent amounts of cDNA in each PCR was verified by amplification of β-actin under similar limiting conditions.

### Immunocytochemistry

Sections (5 μm) were cut from formalin-fixed, paraffin-embedded, tissue sections. The diagnosis in each case was confirmed by examination of haematoxylin and eosin-stained sections. For immunocytochemistry, sections were pressure-cooked in citrate buffer, then stained with antibodies: p14^ARF^ goat polyclonal (C20, Santa Cruz, SC-8613) was used at 1/100 dilution; p73 mouse monoclonal antibody (Neomarkers, MS-764-P0) affinity-purified and diluted 1/150. Sections were scored independently by at least two pathologists.

## RESULTS

### p73 is deregulated by HPV 16E7

**Figure 1:**
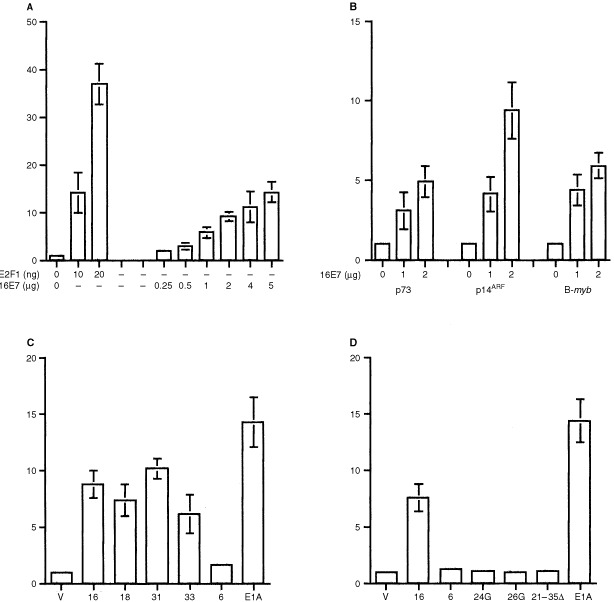
The p73 promoter is activated by HPV E7 proteins in H1299 cells. (**A**) Cells were transfected with p73 luciferase, and the indicated amounts of either pcDNA E2F1 or pcDNA3 16E7. Luciferase activity was determined after 48 h as described in Materials and Methods. Data shown are means of luciferase activity relative to cells receiving only empty vector ±s.d. of at least eight experiments. (**B**) Activation of the p73, p14^ARF^ and B-*myb* promoters by HPV 16E7. H1299 cells were transfected with the indicated luciferase reporter plasmids, and the indicated, increasing amounts of pcDNA3 16E7. Luciferase activity was determined after 48 h as described in Materials and Methods. Data shown are means of relative luciferase activity ±s.d. of at least six experiments. (**C**) Activation of the p73 promoter by E7 proteins from ‘oncogenic’ HPV types. Cells were transfected with p73 luciferase and pcDNA E7 plasmids from the indicated HPV types or empty vector alone (V). Luciferase activity was determined after 48 h as described in Materials and Methods. Data shown are means of relative luciferase activity ±s.d. of at least six experiments. (**D**) Activation of p73 is abrogated by mutants lacking binding to pRb. Cells were transfected with p73 luciferease and the indicated HPV E7 expression plasmids or empty vector only (V). Luciferase activity was determined after 48 h as described in Materials and Methods. Data shown are means ±s.d. of at least six experiments

Analysis of the effect of expression of HPV 16E7 on the activity of the p73 promoter was performed in transient assays in human H1299 cells in which the *RB* gene is intact. Consistent with previous studies, E2F1 strongly activates the p73 promoter (Figure 1A

). Expression of HPV 16E7 results in a dose-dependent increase in activity from the p73 promoter (Figure 1A). The increase in activity from the p73 promoter was comparable to that observed from the promoters of two other recognized E2F1-responsive genes, p14^ARF^ and B-*myb*, in parallel transfections (Figure 1B). We were next interested to determine whether deregulation of p73 was restricted to oncogenic HPV types associated with cervical cancer and high-grade pre-neoplastic lesions. We therefore analyzed the ability of E7 proteins of ‘high-risk’ HPV types (16, 18, 31 and 33) and the ‘low-risk’ HPV 6 to activate expression from the p73 promoter. These studies revealed that activation of p73 occurred with significantly greater efficiency in the case of the oncogenic HPV types, whereas this activity was virtually undetectable in HPV 6 E7 (Figure 1C). As previously reported (Zaika *et al*, 2001), adenovirus E1A also activated the p73 promoter (Figure 1C). One of the differences between the high and low risk E7 proteins is the ability to complex with pRb (Munger *et al*, 1989). We investigated the potential role of this interaction by analysis of a series of mutants of HPV 16E7 (Edmonds and Vousden, 1989). As observed previously, expression of wild-type HPV16 E7 reproducibly results in increased activity from the p73 promoter, but mutants E7Δ21-35 and E7 Gly 24, which do not associate with pRb, failed to increase activity from the p73 promoter (Figure 1D). E7 Gly 26, which fails to associate with pRb yet retains binding to p107, also fails to transactivate p73. These results are consistent with deregulation of p73 expression by HPV 16E7 via E2F1 through association with pRb. Consistent with this hypothesis, expression of HPV 16E7 in the Saos-2 cell line, which lacks endogenous pRb, failed to transactivate the p73 promoter (data not shown).

### HPV E7 activates expression of p73 in human keratinocytes

**Figure 2:**
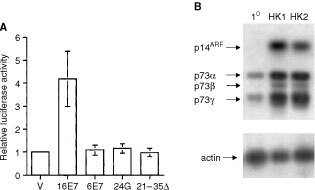
Expression of p73 is activated by HPV 16E7 in human keratinocytes. (**A**) Primary human keratinocytes were transfected with p73 luciferease and the indicated pcDNA HPV E7 expression plasmids. Luciferase activity was determined after 48 h as described in Materials and Methods. Data shown are means±s.d. of at least three experiments. (**B**) Steady-state levels of p14^ARF^ and 73 mRNA in keratinocyte cell lines expressing HPV 16 E7. Lanes are: 1^0^=primary keratinocytes, HK1 and HK2=keratinocyte cell lines immortalized by HPV16 E6 and E7. RT–PCR and hybridization was performed as described in Materials and Methods.

To verify the results obtained in transient assays in H1299 cells, we performed similar analysis in primary cultures of human keratinocytes. In agreement with the data from H1299, HPV 16E7 activated expression from the p73 promoter, albeit at lower levels than observed in H1299, whereas HPV 6E7, 16E7 Gly24 and 16E7 21-35Δ lacked this activity (Figure 2A

). Having observed activation of p73 by oncogenic HPV types in transient assays, we next determined whether steady-state levels of p73 RNA are elevated in keratinocyte cell lines expressing HPV 16E7. In primary keratinocytes, a number of isoforms of full-length p73 were identified by RT–PCR, consistent with previous studies (De Laurenzi *et al*, 1998), although the principal variants were p73α and p73γ (Figure 2B). In two keratinocyte cell lines which stably express HPV 16E7, there is clearly elevated expression of both full-length p73α, p73β and p73γ relative to primary keratinocytes (Figure 2B).

### p73 is deregulated as full-length and* Δ*2 variants in anogenital neoplasia

**Figure 3:**
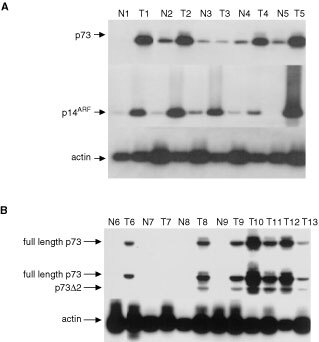
Expression of p14^ARF^and p73 in cervical epithelium. RT–PCR was performed as described in Materials and Methods. (**A**) p73 is over-expressed in HPV 16 positive cervical SCC. A single variant of p73 (p73α) is predominantly expressed in both normal (N) and malignant (T) cervical epithelium. The presence of equivalent amounts of cDNA in each sample was verified by amplification of β-actin as shown. (**B**) Cervical SCC express both full-length and Δ2 forms of p73. RT–PCR for p73Δ2 was performed as described in Materials and Methods. Amplification products were resolved on 2% agarose gels, then hybridized with oligonucleotide probes derived from exon 2 (upper blot) or exon 3 (lower blot). Hybridization with the probe from exon 2 detects only the full-length transcript, whereas the probe derived from exon 3 detects both the full-length and Δ2 forms of p73. In all cases N=normal tissue, T=tumour. Lanes N6 to T9 are four matched cases of normal and tumour. Lanes 10–13 are four examples of cervical SCC, shown without matched normal tissue. The presence of equivalent amounts of cDNA in each reaction was confirmed by amplification of β-actin as shown in the lower panel.

To address the relevance of the results obtained from transient assays and cell lines, we performed RT–PCR and immunocytochemical analysis of p73 expression in primary cervical tissues, these comprising a series of HPV 16 positive CIN I, CIN III and cervical SCC (Figures 3

and 4

**Figure 4:**
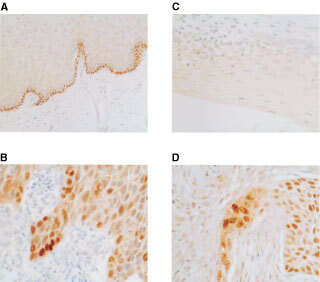
Immunocytochemical analysis of p73 and p14^ARF^ in cervical epithelium. Sections were prepared from formalin-fixed, parrafin- embedded tissue samples as described in Materials and Methods. (**A**) Expression of p73 is in the basal layer of normal cervical epithelium. RT–PCR analysis revealed that this was full-length, p73α in all cases studied. (**B**) p73 protein is over-expressed in cervical SCC. RT–PCR analysis revealed expression of both full-length and p73 Δ2 in the majority of cases. (**C**) p14^ARF^ protein expression is not detectable in normal cervical epithelium. (**D**) Over-expression of p14^ARF^ in cervical SCC. Note the prominent nucleolar expression and sparing of normal tissue.

). Using cycling conditions from the exponential portion of the PCR amplification curve, expression (predominantly of the full-length α form) was detectable in 11 out of 78 samples of normal cervical epithelium. Analysis of cervical intra-epithelial neoplasia (CIN) I samples also failed to detect p73 mRNA in the majority of cases under similar conditions. In contrast, although expression in cervical SCC is also restricted to the α form in almost all cases, steady-state levels of p73 mRNA are markedly increased relative to matched normal tissue in 64 out of 78 (82%) of the cases analyzed (Figure 3A). Elevated expression was observed in nine out of 14 (64%) cases of CIN III, in each case only the α variant being detected. To determine whether samples apparently negative for p73 under these limiting conditions actually expressed low levels of mRNA, PCR was repeated using 40 rather than 28 cycles. Under such conditions, p73 RNA was detected in 72 out of 78 of the normal samples analyzed and eight out of eight of the CIN I samples. This result is consistent with immunocytochemical detection of p73 protein (see below).

The over-expression of p73α in a high proportion of cases of cervical neoplasia was somewhat unexpected in view of the recognized apoptotic and negative growth regulatory functions of p73. We therefore performed additional RT–PCR analyses to examine the N-terminus of the p73 mRNA species expressed in cervical tissues. In these experiments we detected over-expression of the Δ2 variant in the majority of cervical SCC shown in previous analyses to over-express p73α (Figure 3B). There was simultaneous over-expression of full-length p73 in approximately 50% of the cervical SCC. Immunocytochemical analysis confirmed that the increased levels of p73 mRNA revealed by RT–PCR in neoplastic epithelium were reflected in protein expression (Figure 4). Expression of p73 was detected in each of the normal epithelial samples analyzed, consistent with RT–PCR analyses performed under extended cycling conditions, but was restricted to the basal and suprabasal layers in all cases analyzed. Expression increased with increasing grade in CIN. In contrast to normal epithelium, expression was not restricted to the basal and suprabasal layers but was detectable throughout the neoplastic epithelium (Figure 4).

### p73 expression is deregulated with p14^ARF^ in anogenital neoplasia

Finally, we determined the expression levels of another recognised E2F1 regulated gene, p14^ARF^ (Bates *et al*, 1998) in the same samples analysed for p73. In 62 out of 74 cervical cancers (84%) and 11 out of 14 CIN III (79%), steady-state levels of p14^ARF^ mRNA were clearly elevated relative to matched normal tissue (Figure 3A). There was simultaneous deregulation of both p73α and p14^ARF^ in the majority of cases. Immunocytochemical analysis revealed that, in agreement with RT–PCR analyses, p14^ARF^ expression was low to undetectable in normal cervical epithelium (Figure 4), but was abundantly expressed in almost all cases of both cervical SCC and CIN III examined and was over-expressed with p73 in the great majority of cases. The frequent simultaneous over-expression of p14^ARF^ with p73 is consistent with E2F1-driven expression of both genes.

## DISCUSSION

In this work we present data suggesting that functional inactivation of pRb via interaction with HPV 16E7 results in p73 over-expression in transient assays and in cell lines expressing E7 from oncogenic HPV types. We extend these *in vitro* observations by demonstrating, at mRNA and protein levels, deregulated expression of p73 in a high proportion of cervical cancers and pre-malignant lesions.

Initial studies in H1299 cells confirmed the work of other authors showing E2F1 transactivation of the human p73 promoter (Irwin *et al*, 2000; Lissy *et al*, 2000), consistent with the presence of E2F1 consensus binding sites in the p73 promoter (Ding *et al*, 1999). In conjunction with the subsequent observation that E7-dependent activation of the p73 promoter is lost in E7 mutants unable to associate with pRb, these results imply that E7-dependent transactivation of p73 occurs through E2F1. The strong association between deregulated expression of p14^ARF^ and p73 in the CIN III and SCC is clearly consistent with E2F1-dependent activation of expression. Taken together, these results all favour the hypothesis that expression of E7 from oncogenic HPV types results in E2F1-dependent activation of p73 expression. The ability of E7 proteins from oncogenic HPV types to activate p73 is further evidence of functional homologies with E1A and *myc*, since these oncoproteins also deregulate p73 expression (Zaika *et al*, 2001).

p73 is over-expressed in a number of cancers, for example, in 40% of bladder carcinomas (Chi *et al*, 1999), and in breast adenocarcinomas (Zaika *et al*, 1999), although the mechanism underlying over-expression has not been elucidated. In these and other studies, a number of isoforms of p73 generated by alternative splicing of exons encoding the −COOH terminus of the protein were described (De Laurenzi *et al*, 1998). Keratinocytes derived from normal skin also express numerous variants (De Laurenzi *et al*, 1998), in agreement with the present work. It is of interest, therefore, that p73 expression is predominantly of the α isoform in almost all cases of both normal and neoplastic cervical epithelium we have studied. Immunocytochemical analysis revealed that expression of p73 is restricted to the basal and supra-basal layers in normal cervical epithelium, but becomes far more widespread in both CIN and in SCC. In many cases, expression occurs throughout the cancer. We have also observed a striking increase in expression of p73 with increasing grade of neoplasia.

p73 contributes to the apoptotic function of E2F1 (Irwin *et al*, 2000; Lissy *et al*, 2000). In view of this observation and the hypothesized role of p73 as a tumour suppressor protein, it was surprising to observe over-expression in such a high-proportion of cases and to demonstrate a relationship between increasing grade of neoplasia and expression of p73. Sequencing revealed that mutations in p73 are extremely rare in cervical cancers (data not shown), consistent with other authors' studies of both solid and haematological malignancies. p73 Δ2, which lacks exon 2 of p73, was recently described in ovarian carcinomas (Ng *et al*, 2000) and breast cancer cell lines (Fillippovich *et al*, 2001). p73Δ2 is able to inhibit the transactivating functions of both p53 and p73 and, as such, over-expression of this protein represents a potential mechanism by which the tumour suppressor properties of these proteins might be negated or inhibited (Fillippovich *et al*, 2001). These authors suggested that analysis of expression of p73Δ2 in a range of normal and malignant tissues would have considerable interest. We now demonstrate that this variant is frequently over-expressed in cervical SCC and this may, at least in part, be the basis by which the functions of full-length p73α are nullified in such cases. Moreover, there is expression of wild-type p53 protein in a high proportion of cervical SCC (Troncone *et al*, 1998). The ability to inhibit the function of p53 as well as full-length p73 (Fillippovich *et al*, 2001) implies that expression of p73Δ2 may co-operate with HPV E6 to neutralize p53 in cervical neoplasia. Furthermore, because full-length p73α functions in pathways of keratinocyte differentiation, expression of p73Δ2 may also have inhibitory effects on squamous differentiation. Analysis of p73Δ2 expression in squamous cancers of varying differentiation status would clearly be of interest. The high frequency of over-expression of p73 and p14^ARF^ in both cervical SCC and CIN, taken together with the observation of increasing expression with higher grades of neoplasia, raises the possibility that immunocytochemical analysis of these proteins may have utility in diagnostic and/or prognosis in cervical neoplasia. Antibodies specifically detecting p73Δ2 are not yet available. Discrimination between full-length and p73Δ2 would further allow definition of the role of over-expression of these proteins in squamous neoplasia and their potential use as markers of cervical neoplasia.

## References

[bib1] BatesSPhillipsACClarkePAStottFPetersGLudwigRLVousdenKH1998 p14^ARF^ links the tumour suppressors RB and p53 Nature 395 124125 9744267 10.1038/25867

[bib2] ChiS-GChangS-GLeeS-JLeeC-HKimJIParkJ-H1999 Elevated and biallelic expression of p73 is associated with progression of human bladder cancer Cancer Res 59 27912793 10383132

[bib3] CornPGKuerbitzSJvan NoeselMMEstellerMCompitelloNBaylinSBHermanJG1999 Transcriptional silencing of the p73 gene in acute lymphoblastic leukemia and Burkitt's lymphoma is associated with 5′CpG methylation Cancer Res 59 33523356 10416592

[bib4] De LaurenziVRossiATerrinoniABarcoroliDLevreroMCostanzoAKnightRAGuerrieriPMelinoG2000 p63 and p73 transactivate differentiation gene promoters in human keratinocytes Biochem Biophys Res Commun 273 342346 10873608 10.1006/bbrc.2000.2932

[bib5] De LaurenziVCostanzoABarcaroliDTerrinoniAFalcoMAnnicchiarico-PetruzzelliMLevreroMMelinoGJ1998 Two new splice variants, gamma and delta, with different transcriptional activity J Exp Med 188 17631768 9802988 10.1084/jem.188.9.1763 PMC2212516

[bib6] DingYInoueTKamiyamaJTamuraYOhtani-FujitaNIgataESakaiT1999 Molecular cloning and functional characterization of the upstream promoter region of the human p73 gene DNA Res 6 347351 10574463 10.1093/dnares/6.5.347

[bib7] EdmondsCVousdenKH1989 A point mutational analysis of human papillomavirus type 16 E7 protein J Virol 63 26502656 2542578 10.1128/jvi.63.6.2650-2656.1989 PMC250749

[bib8] FillippovichISorokinaNGateiMHauptYHobsonKMoallemESpringKMouldMMcGuckinMALavinMFKhannaKK2001 Transactivation-deficient p73α (p73Delta exon 2) inhibits apoptosis and competes with p53 Oncogene 20 514522 11313982 10.1038/sj.onc.1204118

[bib9] GazzeriSDella ValleVChaussadeLBrambillaCLarsenCJBrambillaE1998 The human p19^ARF^ protein encoded by the beta transcript of the p16^INK4a^ gene is frequently lost in small cell lung cancer Cancer Res 58 39263931 9731504

[bib10] Hawley-NelsonPVousdenKHHubbertNLLowyDRSchillerJT1989 HPV16 E6 and E7 proteins cooperate to immortalize human foreskin keratinocytes EMBO J 8 39053910 2555178 10.1002/j.1460-2075.1989.tb08570.x PMC402081

[bib11] IrwinMMarinMCPhillipsACSeelanRSSmithDILiuWFloresERTsaiKYJacksTVousdenKHKaelinJrWG2000 Role for the p53 homologue p73 in E2F-1-induced apoptosis Nature 407 645648 11034215 10.1038/35036614

[bib12] JostCAMarinMCKaelinJrWG1997 p73 is a simian [correction of human] p53-related protein that can induce apoptosis Nature 389 191194 9296498 10.1038/38298

[bib13] KaghadMBonnetHYangACreancierLBiscanJCValentAMintyAChalonPLeliasJMDuMontXFerraraPMcKeonFCaputD1997 Monoallelically expressed gene related to p53 at 1p36, a region frequently deleted in neuroblastoma and other human cancers Cell 90 809819 9288759 10.1016/s0092-8674(00)80540-1

[bib14] LamELamEWMorrisJDDaviesRCrookTWatsonRJVousdenKH1994 HPV16 E7 oncoprotein deregulates B-myb expression: correlation with targeting of p107/E2F complexes EMBO J 13 871878 8112300 10.1002/j.1460-2075.1994.tb06330.x PMC394886

[bib15] LissyNADavisPKIrwinMKaelinWGDowdySF2000 A common E2F-1 and p73 pathway mediates cell death induced by TCR activation Nature 407 642645 11034214 10.1038/35036608

[bib16] MungerKWernessBADysonNPhelpsWCHarlowEHowleyP1989 Complex formation of human papillomavirus E7 proteins with the retinoblastoma tumor suppressor gene product EMBO J 8 40994105 2556261 10.1002/j.1460-2075.1989.tb08594.x PMC401588

[bib17] NgS-WYiuGKLiuYHuangL-WPalnatiMJunSHBerkowitzRSMokSC2000 Analysis of p73 in human borderline and invasive ovarian tumor Oncogene 19 18851890 10773878 10.1038/sj.onc.1203512

[bib18] StoreyAPimDMurrayAOsbornKBanksLCrawfordL1989 Comparison of the in vitro transforming activities of human papillomavirus types EMBO J 7 18151820 10.1002/j.1460-2075.1988.tb03013.x PMC457173 2458921

[bib19] TronconeGMartinezJCPalombiniLDe RosaGMugicaCRodriguezJAZeppaPDi VizioDLucarielloAPirisMA1998 Immunohistochemical expression of mdm2 and p21Waf1 in invasive cervical cancer: correlation with p53 protein and high risk HPV infection J Clin Pathol 51 754760 10023338 10.1136/jcp.51.10.754 PMC500930

[bib20] WalboomersJMJacobsMVManosMMBoschFXKummerJAShahKVSnijdersPJPetoJMeijerCJMunozN1999 Human papillomavirus is a necessary cause of invasive cervical cancer worldwide J Pathol 189 13 10451482 10.1002/(SICI)1096-9896(199909)189:1<12::AID-PATH431>3.0.CO;2-F

[bib21] ZaikaAIKovalevSMarchenkoNDMollUM1999 Overexpression of the wild type p73 gene in breast cancer tissues and cell lines Cancer Res 59 32573263 10397274

[bib22] ZaikaAIrwinMSansomeCMollUM2001 Oncogenes induce and activate endogenous p73 protein J Biol Chem 276 1131011316 11115495 10.1074/jbc.M005737200

